# A rare case of factor X deficiency induced by valproic acid

**DOI:** 10.1016/j.rpth.2025.102721

**Published:** 2025-03-03

**Authors:** Pierre-Antonin Rigon, Vincent Ernest

**Affiliations:** 1 Aix-Marseille Université, Assistance Publique Hôpitaux de Marseille, Hôpital Conception, Centre de Néphrologie et Transplantation Rénale, Marseille, France; 2 Laboratoire d'Hématologie, Hôpital de la Timone, Assistance Publique-Hôpitaux de Marseille, Marseille, France

**Keywords:** blood coagulation, blood coagulation disorders, drug-related side effects and adverse reactions, factor X, factor X deficiency, valproic acid

## Abstract

**Background:**

Factor X (FX) deficiency (FXD) significantly disrupts coagulation, potentially leading to severe bleeding. While inherited FXD is rare, with a prevalence of 1 in 500,000, acquired FXD is also uncommon and frequently linked to conditions such as light-chain amyloidosis. In rare cases, certain medications can cause FXD.

**Key Clinical Question:**

Here, we present a rare case of acquired FXD induced by valproic acid (VPA). This deficiency is associated with the presence of anti-FX antibodies.

**Clinical Approach:**

A 65-year-old man undergoing treatment for various conditions, including chronic kidney disease and type 2 diabetes, developed severe FXD (activity <2 U/L) following VPA administration for epilepsy. During FXD, the patient experienced significant bleeding episodes, necessitating FX replacement with prothrombin complex concentrate. Upon discontinuation of VPA, FX activity improved in 9 days, possibly suggesting a role of the drug in FXD. Interestingly, antibodies directed against FX have been identified.

**Conclusion:**

This case emphasizes the necessity for clinicians to be vigilant of hemostasis disorders associated with VPA, even though such occurrences are rare.

## Essentials

• AFXD is a rare coagulation disorder.
• We report a case of AFXD induced by sodium valproate medication.
• A patient developed anti-FX antibodies, resulting in very low FX activity.
• AFXD resolved with discontinuation of sodium valproate.

## 1 Introduction

The coagulation process is driven by a series of molecular interactions on endothelial surfaces. One of these, factor (F)X, is of particular interest. Indeed, it plays a central role in the common pathway leading to the formation of thrombin (activated FII) [1]. FX deficiency (FXD) may result in a severe condition as it inactivates the coagulation pathway, potentially causing a major bleed in affected patients [2]. As in other factor deficiencies, FXD can be inherited or acquired. Constitutional FXD is a rare bleeding disorder of autosomal recessive inheritance with a prevalence of the homozygous form evaluated around 1:500,000 [3]. Acquired FXD (AFXD) is rare as well. Amyloid light-chain (AL) amyloidosis is the leading attributor to AFXD through the immunoadsorption of FX by amyloid fibrils [4]. Nonamyloid AFXD is also an exceptional clinical scenario, with less than 60 cases reported in a recent literature review [5]. It can result from the presence of an anti-FX antibody or autoimmune FXD (AiFXD) [6]. Typically, there is an underlying condition associated with nonamyloid AFXD, of which infections (particularly respiratory) are most frequently reported [7]. Though exceptional, some case reports have attributed AFXD to meropenem [8] or sodium valproate [9].

Here, we report the case of a patient presenting with sodium valproate-induced FXD.

## 2 Case Report

A 65-year-old man was admitted to the nephrology department in March 2024 for renal embolization. His medical background consisted of chronic kidney disease due to autosomal dominant polycystic kidney disease. He also presented with arterial hypertension, ischemic cardiomyopathy (requiring a stent insertion 4 months prior), ischemic stroke (2012), and type 2 diabetes. More recently, his medical history was marked by severe recurrent cystic bleeding, which was followed by the decision to embolize one of the kidneys. At admission, his coagulation profile was normal (prothrombin time [PT] 8.2 seconds, activated partial thromboplastin time [aPTT] 25 seconds). The normal range of PT and aPTT in our laboratory is 8.3 to 10 and 20 to 30 seconds, respectively. Embolization was complicated by the occurrence of an ischemic stroke 3 days after and a seizure 2 days after the stroke. Levetiracetam medication was introduced for seizure prophylaxis. However, the patient had another epileptic seizure 14 days later. A decision was made to introduce valproic acid (VPA; Depakine; Sanofi) with a reducing regimen of levetiracetam. His daily medication consisted of bisoprolol, clopidogrel, aspirin, dulaglutide, amlodipine, atorvastatin, and ramipril. Thirty-six hours after the introduction of VPA, the patient presented with minor epistaxis followed by spontaneous resolution. A coagulation assessment was performed, which showed a severe FXD below the 2 U/L limit of quantification. It resulted in an augmented PT of 40.7 seconds. Activities of FII, FV, and FVII were >100 U/L (108 U/L, >150 U/L, and >150 U/L, respectively). It is important to note that the liver test was within normal limits according to cytolysis and cholestasis. The previous coagulation assessment was normal 14 days prior (PT 8.9 seconds, aPTT 25 seconds). The PT assay was performed with the Dade Innovin reagent (Siemens Healthineers). FX activity was measured by a PT-based assay using FX-depleted plasma (Factor X Deficient, Siemens Healthineers) and Dade Innovin. Both assays were conducted according to the manufacturer’s instructions. Normal ranges (70%-120%) were provided by the manufacturer.

**Figure 1:**
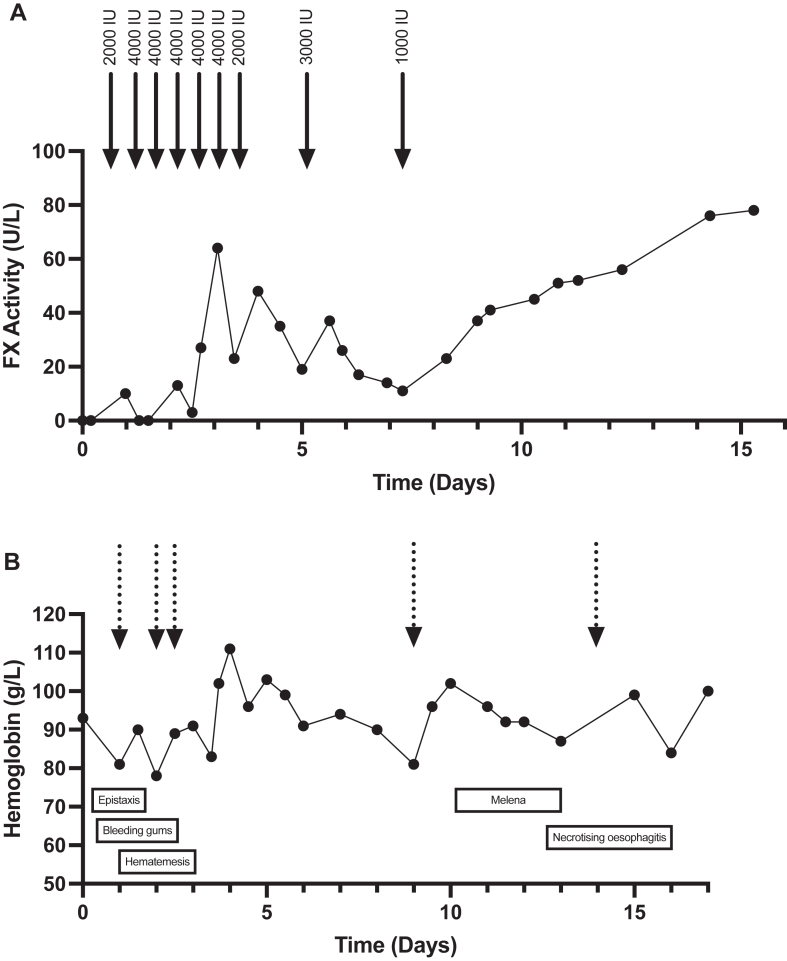
(A) Evolution of factor X (FX) activity over time following prothrombin complex concentrate infusion. (B) Evolution of hemoglobin levels during the FX deficiency episode, alongside the timeline of red blood cell transfusions. Solid arrows indicate prothrombin complex concentrate infusions; the injected dose is shown above the arrow. Dotted arrows represent the transfusion of 2 units of red blood cell concentrate. IU, International Units.

Considering these results, VPA was rapidly discontinued. Acquired von Willebrand syndrome was ruled out (von Willebrand factor [VWF] activity > 394 U/L). Due to clinical bleeding complications (hemorrhagic transformation of the recent ischemic stroke, epistaxis, and bleeding gums), a FX replacement using prothrombin complex concentrate (PCC; Octaplex; Octapharma) was initiated; plasma-derived FX concentrates were unavailable for acquired deficiencies. Being aware of the thrombotic risk of PCC in this patient, especially regarding his recent coronary stent insertion, we aimed to obtain 20 U/L of residual FX activity. We managed this delicate balance by measuring his coagulation profile before and after PCC infusion twice a day. The evolution of FX activity and PCC infusion is shown in Figure 1. Initially, 4000 International Units (IU) of PCC were administered. If, 12 hours later, the coagulation test showed an activity level of <10 U/L, a 4000 IU dose was given. If the activity was between 10 and 20 U/L, a 3000 IU dose was administered. No administration was required if the activity exceeded 20 U/L. Using this protocol, the duration of replacement was 9 days, with a total of 28,000 IU of PCC administered. During this episode, the patient experienced major bleeding, such as recurrent epistaxis, gastrointestinal bleeding secondary to necrotizing esophagitis, and macroscopic hematuria. Ten red blood cell units were transfused. Hemoglobin variation and red blood cell concentrate transfusion are also shown in Figure 1. PCC administrations were progressively reduced and were discontinued after 9 days. A coagulation profile performed 1 month after AFXD showed a PT of 10.3 seconds and FX activity of 89 U/L. All the hemostasis assessments are presented in the Supplementary Table. The patient also received imipenem and linezolid for cyst infection during this admission. However, FXD resolved without the discontinuation of these treatments, making their involvement unlikely.

**Figure 2:**
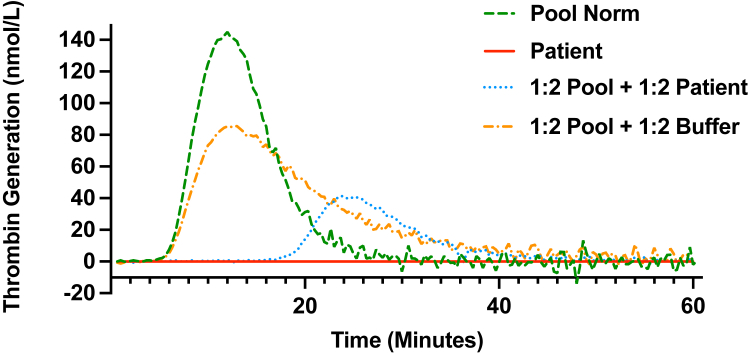
Thrombin generation assays using Pool Norm (Stago) and plasma from the patient and mixing tests using the patient’s plasma or Owren’s buffer in addition to Pool Norm (Platelet-Poor Plasma Reagent low, tissue factor 1 pM, Stago). Curves represent peak heights at different times during mixing tests using 1:2 patient plasma or 1:2 buffer and 1:2 Pool Norm.

To further investigate this severe AFXD, some complementary laboratory tests were performed. We used thrombin generation assays on the patient’s plasma and mixing tests, adding control plasma to the patient’s plasma or a buffer solution using 1:1 or 1:3 dilution ratios (Figure 2). Thrombin generation was measured in plasma prepared as follows: 20 μL of working buffer containing 4 μM of phospholipid with 1 pM of tissue factor (Bleedscreen Reagent, Diagnostica Stago) was placed in 96-well plates, and 80 μL of plasma or mixed-plasma was added to each well. Mixing tests used the patient’s plasma, Pool Norm (Diagnostica Stago), and Owren’s buffer (Diagnostica Stago). Thrombin generation was initiated by adding 20 μL of fluorogenic substrate/CaCl_2_ buffer (FluCa Kit, Diagnostica Stago). Fluorescence was measured using a fluorometer (Fluoroskan Ascent, Thermolab Systems) with an excitation filter at 390 nm and an emission filter at 460 nm. Samples were tested in duplicate. Thrombin generation curves were analyzed for lag time (minutes), peak height (nmol/L), and endogenous thrombin potential (nmol/L.min) using dedicated software (Thrombinoscope). Tested alone, the patient’s plasma displayed no thrombin generation. Mixing tests showed reduced thrombin generation when the patient’s plasma was added to control Pool Norm compared with thrombin generation of mixed Pool Norm and Owren’s buffer, which contains no coagulation factor. Lag time, peak height, and endogenous thrombin potential during thrombin generation assay are shown in the Table. These results were in favor of the presence of an inhibitor of coagulation in the plasma of our patient. To demonstrate the presence of this inhibitor, mixing tests and an enzyme-linked immunosorbent assay (ELISA) were performed. We performed mixing and diagnostic tests for a lupus anticoagulant using Stago PTT-LA Reagent (Diagnostica Stago). The patient’s plasma clotting time was 172.3 seconds, whereas the control plasma clotting time was 34 seconds. The clotting time of the mixture (50:50) was 63.7 seconds. Failure of correction suggested the presence of an inhibitor. Diagnostic testing for lupus anticoagulant employed Stago PTT-LA Reagent (Diagnostica Stago) for screening. For mixing tests, patient plasma was mixed with Pool Norm (Diagnostica Stago) at a ratio of 1:1. Control Pool Norm (Diagnostica Stago) FX activity was measured at 101 U/L. Concerning the ELISA, after several dilutions, it revealed the presence of an anti-FX immunoglobulin G antibody, with a high titer of 1:640 (maximum dilution).

**Table:** Lag time, peak, and endogenous thrombin potential during thrombin generation assay according to a mixture between the patient’s plasma, pooled plasma (Pool Norm), and Owren’s buffer.

Parameters	Pool Norm	Patient plasma	1:2 pool + 1:2 plasma	1:2 pool + 1:2 buffer
**Lag time (min)**	6.67	0	19	6.67
**Peak (nmol/L)**	143.19	0	40.96	85.39
**ETP (nmol/L.min)**	1333	0	489.5	1378

ETP, endogenous thrombin potential.

Given the known association with AL amyloidosis, we performed plasmatic protein electrophoresis. It resulted in 2 peaks of immunoglobulin G kappa and lambda, with low values of 0.5 and 0.2 g/L, respectively. Light chains were additionally analyzed and revealed a well-balanced state (kappa/lambda ratio, 0.91). Thus, the presence of AL amyloidosis was judged as unlikely. Unfortunately, we failed to measure VPA concentration in plasma.

## 3 Discussion

We report the case of a 65-year-old man diagnosed with AFXD associated with the treatment of VPA. Since its introduction in the 1960s [10], VPA has gained widespread use and holds a prominent role in recent clinical guidelines [11]. However, its use is not without risks, as it is associated with a variety of adverse events. The most frequently reported complications include hepatotoxicity, hyperammonemia encephalopathy, and endocrine effects such as weight gain and hyponatremia [12]. VPA has also been linked to neurodevelopmental disorders, including autism [13]. Of particular interest is its effects on hemostasis, which may impair both primary and secondary hemostasis. Primary hemostasis involves the aggregation of platelets at injury sites, with platelets and VWF playing key roles. Secondary hemostasis reinforces platelet aggregation through a sequence of coagulation factor activation [14]. VPA has been associated with disruptions in both primary and secondary hemostasis, as described by Kumar et al. [15] in 2019. Additionally, a study by Post et al. [16] observed hemostasis disorders in 46.6% of patients (34 out of 73) treated with VPA, primarily characterized by platelet dysfunction and thrombocytopenia. The Post et al. [16] study also confirmed the incidence of thrombocytopenia around 10%, as reported by Delgado et al. [17].

The mechanism underlying this induced thrombocytopenia remains poorly understood. It is likely multifactorial, involving both immune-mediated pathways and direct bone marrow toxicity [18,19]. VWF deficiency, FXIII deficiency, and hypofibrinogenemia have also been described [20,21], even if VWF deficiency is inconsistent in the literature [22]. The mechanism is also unclear, suggesting that a part of the hepatotoxicity of VPA is leading to these deficiencies. Importantly, no liver cytolysis or cholestasis was present in our case. A case of isolated FVII deficiency has been reported in a 3-year-old boy treated with VPA. This disorder resolved 12 months after VPA discontinuation [23]. Once again, the mechanism suspected is liver toxicity by VPA, but liver tests remained within normal values in this patient. VPA AFXD is also a rare condition with, to our knowledge, only 1 case reported in the literature [9]. An 85-year-old woman receiving VPA for generalized seizure was hospitalized for alteration of her general condition 1 month after the introduction of VPA. Laboratory testing showed a decreased FX activity of 7% with prolonged PT. FII, FV, and FVII activities and platelet count were normal. No FX inhibitor was detected in the patient’s plasma. She presented with rectal bleeding and metrorrhagia requiring FX concentrate infusion. It resulted in poor clinical and biological improvement, with a FX activity of 23% 2 hours after infusion. Similarly to our patient, VPA was withdrawn, and coagulation parameters returned to normal within 15 days. Interestingly, in our case, an antibody directed to FX was identified by ELISA with a high titer of 1:640. This finding is inconsistent with AiFXD cases. Indeed, as described by Ichinose et al. [5], only 8 of 26 AiFXD cases presented with an antibody directed to FX. The rest of the cases were defined as “probable cases” because they showed the presence of an inhibitor by different tests without revealing any antibody. Furthermore, our patient’s coagulation profiles were consistent with the presence of an antibody. Indeed, PCC contains between 720 and 1200 IU of FX in each bottle. The half-life of FX is expected to be around 30 hours [24]. However, after the infusion, our patient’s FX activity initially increased but then decreased rapidly within <24 hours. This suggests accelerated clearance, potentially caused by the highlighted antibody. In the cases described in the literature, one of the mechanisms proposed to explain this antibody emergence is a potential structural similarity between some viruses or antibiotics and FX [5,7,25]. Concerning VPA, observations are scarce, but a similar mechanism cannot be ruled out. As the patient received several other medications, the causality of these drugs is plausible but less likely to be implicated than VPA. Regarding levetiracetam, although rare, its use has been linked mainly to thrombocytopenia [26], with no mechanism identified to date. Albeit, 1 case of Willebrand disease induced by levetiracetam has been reported [27]. Nevertheless, no AFXD has been reported. Concerning our patient, AFXD resolved without levetiracetam discontinuation, making its involvement unlikely. Unfortunately, no coagulation assessment was performed between levetiracetam and VPA introduction. Concerning other drugs, clopidogrel and aspirin have been associated with acquired hemophilia A but not with AFXD [28,29]. Thrombocytopenia under amlodipine or atorvastatin medication has been described [[30], [31], [32]], and ramipril use can modify platelet aggregation [33]. However, no factor deficiencies have been reported to date. Finally, no previous study has linked bisoprolol or dulaglutide to hemostasis disorders. Given the timing of the occurrence of AFXD and its regression without discontinuation of the other medications, VPA is the most likely drug associated with this AFXD.

To conclude, VPA-associated AFXD is a rare condition. Nevertheless, it underlines the fact that VPA could be implicated in numerous hemostasis disorders and that clinicians must remain vigilant toward its utilization. Numerous studies showed the importance of performing a coagulation assessment after introducing VPA to a patient, especially if surgery is planned.
