# Supplementary material for: A rare case of factor X deficiency induced by valproic acid
Source: Res Pract Thromb Haemost. 2025 Mar 3;9(2):102721. doi: 10.1016/j.rpth.2025.102721 (PMC11981724; doi:10.1016/j.rpth.2025.102721)
Supplement: Supplementary Table [file mmc1.docx]

| **Date** | **5/3/24**  **5:00 AM** | **13/3/24**  **5:00 AM** | **27/3/24**  **10:00 PM** | **28/3/24**  **2:37 AM** | **28/3/24**  **9:30 PM** | **29/3/24**  **5:00 AM** | **29/3/24**  **10:00 AM** | **30/3/24**  **2:00 AM** |
| --- | --- | --- | --- | --- | --- | --- | --- | --- |
| **PT normal range (s)** | 8.3-10 | | | | | | | |
| **aPTT normal range (s)** | 20-30 | | | | | | | |
| **PT patient (s)** | 8.2 | 8.9 | 40 | 48.8 | 25.2 | 56.1 | 65.4 | 26.7 |
| **aPTT patient (s)** | 25 | 25 | 70 | 79 | ND | 83 | ND | 53 |
| **FX activity (U/L)** | *NA* | *NA* | <2 | <2 | 10 | <2 | <2 | 13 |
| **Date** | **30/3/24**  **10:00 AM** | **30/3/24**  **2:50 PM** | **30/3/24**  **11:50 PM** | **31/3/24**  **9:00 AM** | **31/3/24**  **10:00 PM** | **1/4/24**  **10:00 AM** | **1/4/24**  **10:00 PM** | **2/4/24**  **1:10 PM** |
| **PT normal range (s)** | 8.3-10 | | | | | | | |
| **aPTT normal range (s)** | 20-30 | | | | | | | |
| **PT patient (s)** | 51.6 | 19.8 | 14.8 | 22.5 | 16.9 | 20.6 | 26.4 | 19.1 |
| **aPTT patient (s)** | 63 | 44 | ND | 46 | 40 | 43 | 49 | 44 |
| **FX activity (U/L)** | 3 | 27 | 64 | 23 | 48 | 35 | 19 | 37 |
| **Date** | **2/4/24**  **8:00 PM** | **3/4/24**  **5:00 AM** | **3/4/24**  **8:30 PM** | **4/4/24**  **5:00 AM** | **5/4/24**  **5:00 AM** | **5/4/24**  **10:00 PM** | **6/4/24**  **5:00 AM** | **7/4/24**  **5:00 AM** |
| **PT normal range (s)** | 8.3-10 | | | | | | | |
| **aPTT normal range (s)** | 20-30 | | | | | | | |
| **PT patient (s)** | 22.3 | 27.6 | 28 | 28.1 | 19.7 | 14.4 | 13.8 | 13 |
| **aPTT patient (s)** | 45 | 52 | 49 | 56 | 48 | 44 | 41 | 36 |
| **FX activity (U/L)** | 26 | 17 | 14 | 11 | 23 | 37 | 41 | 45 |
| **Date** | **7/4/24**  **6:00 PM** | **8/4/24**  **5:00 AM** | **9/4/24**  **5:00 AM** | **10/4/24**  **11:07 AM** | **11/4/24**  **5:00 AM** | **12/4/24**  **5:00 AM** |  |  |
| **PT normal range (s)** | 8.3-10 | | | | | |  |  |
| **aPTT normal range (s)** | 20-30 | | | | | |  |  |
| **PT patient (s)** | 12.7 | 11.9 | 11.9 | 11.7 | 10 | 9.9 |  |  |
| **aPTT patient (s)** | 35 | 34 | 31 | 30 | 30 | 29 |  |  |
| **FX activity (U/L)** | 51 | 52 | 56 | *NA* | 76 | 78 |  |  |

**Supplementary Table.** Coagulation Assesment during factor X deficiency episode. PT: Prothrombin time, aPTT: activated partial thromboplastin time, FX: Factor X, NA: not available.
